# The N-terminus of *Lactobacillus amylovorus* feruloyl esterase plays an important role in its secretion by *Lactobacillus plantarum* and *Escherichia coli*

**DOI:** 10.1186/s12934-021-01645-9

**Published:** 2021-08-03

**Authors:** Zhenshang Xu, Rongling Zhang, Ting Wang, Jian Kong

**Affiliations:** 1 State Key Laboratory of Biobased Material and Green Papermaking, Qilu University of Technology, Shandong Academy of Science, No.3501, Daxue Road, Jinan, 250353 People’s Republic of China; 2 School of Bioengineering, Qilu University of Technology, Shandong Academy of Science, Jinan, 250353 People’s Republic of China; 3 State Key Laboratory of Microbial Technology, Shandong University, No.72, Binhai Road, Qingdao, 266237 Shandong People’s Republic of China

**Keywords:** Feruloyl esterase, Secretion, N-terminus

## Abstract

**Background:**

Feruloyl esterase is a multifunctional esterase with potential industrial applications. In the present study, we found the *Lactobacillus amylovorus* feruloyl esterase (FaeLam) could be secreted by *L. plantarum* and *Escherichia coli*. However, no signal peptide was detected in this protein as predicted by SignalP-5.0. Therefore, experiments were carried out to propose an explanation for the extracellular release of FaeLam.

**Results:**

Here, we identified that the FaeLam could be secreted to the culture medium of *L. plantarum* CGMCC6888 and *E. coli* DH5α, respectively. To exclude the possibility that FaeLam secretion was caused by its hydrolytic activity on the cell membrane, the inactive FaeLam^S106A^ was constructed and it could still be secreted out of *L. plantarum* and *E. coli* cells. Furthermore, the truncated version of the FaeLam without the N-terminal residues was constructed and demonstrated the importance of the 20 amino acids of N-terminus (N20) on FaeLam secretion. In addition, fusion of heterologous proteins with N20 or FaeLam could carry the target protein out of the cells. These results indicated the N-terminus of FaeLam played the key role in the export process.

**Conclusions:**

We proved the N-terminus of *L. amylovorus* FaeLam plays an important role in its secretion by *L. plantarum* and *E. coli*. To our best knowledge, this is the first reported protein which can be secreted out of the cells of both Gram-positive and Gram-negative bacteria. Furthermore, the results of this study may provide a new method for protein secretion in *L. plantarum* and *E. coli* through fusion the target protein to N20 of FaeLam.

**Supplementary Information:**

The online version contains supplementary material available at 10.1186/s12934-021-01645-9.

## Background

Feruloyl esterases are a subclass of carboxylic acid esterases that hydrolyze the ester bond between hydroxycinnamic acids and sugars [1]. As important auxiliary enzyme for plant biomass degradation, they have been widely used in fuel ethanol production, pulp and paper industries, and animal feed additives [2]. In recent years, feruloyl esterases have aroused interest because of their ability to release hydroxycinnamic acids from plant materials to exert their bioactivity directly. Many studies in vitro and in vivo have indicated that hydroxycinnamic acids (especially ferulic acid) can prevent the oxidation of low-density lipoprotein, inhibit the spread of tumors, and protect against certain chronic diseases such as coronary heart disease and some cancers [3].

Considering the importance in various applications, many feruloyl esterases have been found and isolated from a large number of fungal and bacterial sources [4]. Recently, more and more feruloyl esterases have been identified and characterized in different *Lactobacillus* species, which have generally recognized as safe (GRAS) status and long history of use in food applications [5]. Given the essential role of feruloyl esterase in the production of hydroxycinnamic acids, plus the GRAS status of *Lactobacillus* species, which therefore have less regulatory concerns, feruloyl esterases from probiotic *Lactobacillus* strains can be directly used for increased production of high-value hydroxycinnamates and ferulic acid from natural or synthetic carbon sources [6]. In general, *Lactobacillus* feruloyl esterases are heterologously expressed in host cells and then purified for applications. However, whether feruloyl esterase can be secreted by *Lactobacillus* strains is still unknown [6, 7].

The Gram-negative bacterium *Escherichia coli* is a commonly used cell factory for the production of feruloyl esterases, because it is the best characterized host with many available expression and regulation tools [8, 9]. However, the common laboratory strains of *E. coli* are poor secretors for proteins, resulting from the complex cell envelope with two layers [10]. Unexpectedly, our previous study showed that the high yield of feruloyl esterase (FaeLam derived from *L. amylovorus*) could be secreted from *E. coli* BL21(DE3) with the pET expression system [11]. Despite the several reports about secretory proteins in *E. coli*, there is no conclusive and universally secretion pathway identified so far [12, 13]. A greater understanding of feruloyl esterase secretion has application significance in protein production or therapeutic purposes.

In this study, we reported that the feruloyl esterase FaeLam from *L. amylovorus* CGMCC11056 could be secreted from *L. plantarum* CGMCC6888 and *E. coli* DH5α respectively when heterologously overexpressed by *Lactobacillus*/*E. coli* shuttle vector. In order to further explore this anomalous secretory phenomenon, the inactive mutant and truncated mutants of FaeLam was constructed to assess the activity or sequence requirements for secretion. Moreover, the ability of FaeLam and its N-terminal sequence as carriers to export protein was also performed.

## Results

### Construction of the shuttle vector pLP3804

**Fig. 1:**
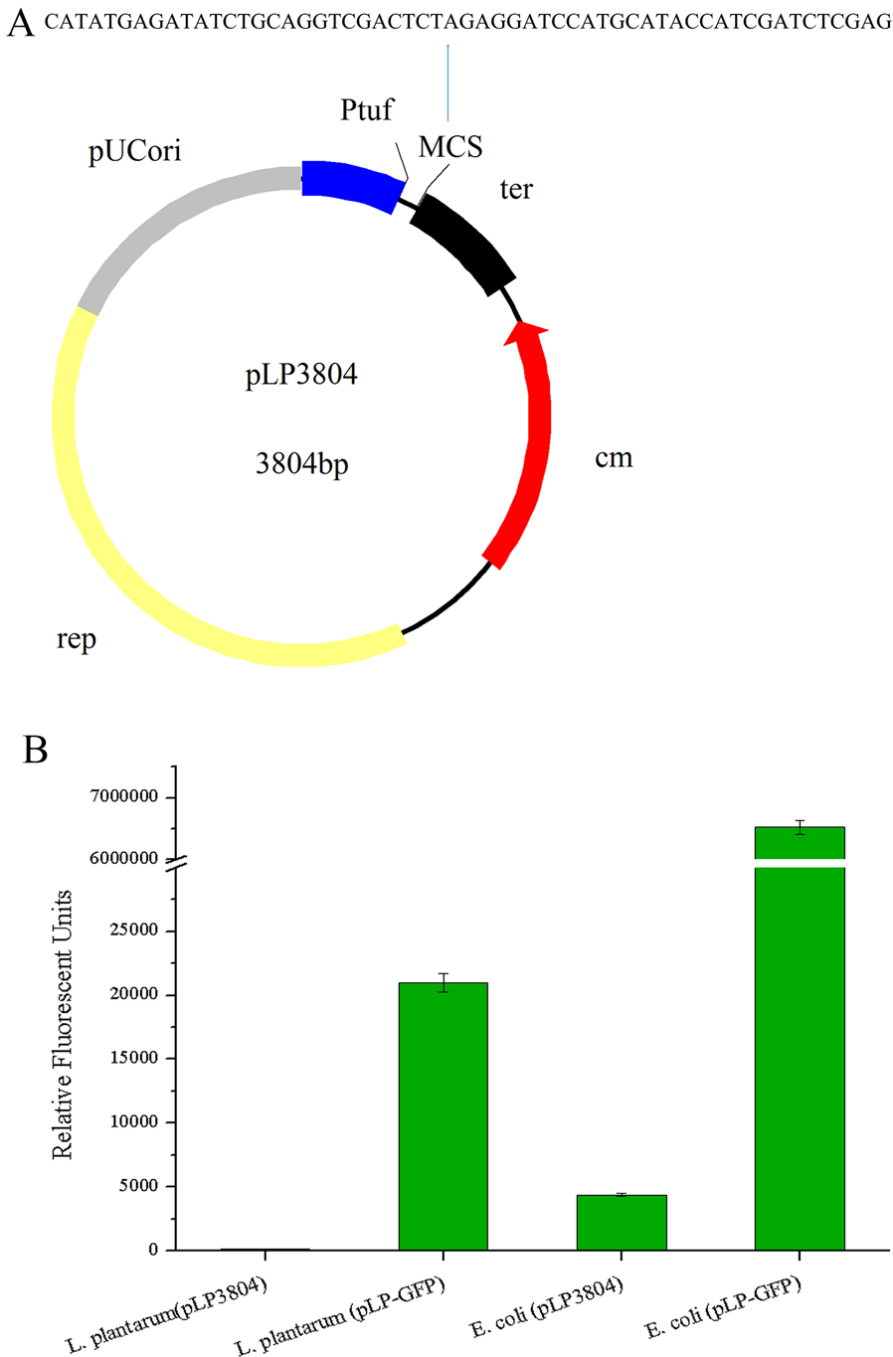
The construction of vector pLP3804. **A** Map of the *Lactobacillus*/*Escherichia coli* shuttle expression vector of pLP3804. The multiple cloning sites are showed in expanded view. **B** The relative fluorescent units of the recombinant *L. plantarum* CGMCC6888 and *E. coli* DH5α cells expressing green fluorescent protein

To investigate the expression and secretion of FaeLam and its mutants in *L. plantarum* and *E. coli*, a *Lactobacillus*/*E. coli* shuttle vector named pLP3804 was constructed. The detailed composition of the expression vector pLP3804 was presented in Fig. 1A. This vector had a promoter P_tuf_, which had been verified to be able to express protein in *L. plantarum* constitutively [14]. Furthermore, the first restriction enzyme site contained in the multiple cloning sites was CATATG, which allowed the heterologous proteins to be expressed with a natural N-terminus when restriction enzyme *Nde*I was applied. Green fluorescence protein (GFP) derived from vector pMN402 was used as the reporter protein to evaluate the feasibility of pLP3804 for heterologous protein expression in *L. plantarum* and *E. coli* [15]. The *gfp* gene was cloned into pLP3804 and the resultant plasmid pLP-GFP was transformed into competent cells of *L. plantarum* CGMCC6888 and *E. coli* DH5α. The green fluorescence in recombinant cells was observed (Fig. 1B), while no fluorescence was observed in control strains (*L. plantarum* CGMCC6888 and *E. coli* DH5α with pLP3804), suggesting that the promoter P_tuf_ could initiate protein expression not only in *L. plantarum* but also in *E. coli*. SDS-PAGE was carried out to confirm the expression level of GFP. The results showed that one more protein band clearly appeared in the *E. coli* harboring pLP-GFP (Additional file 1: Figure S1). This phenomenon was consistent with the difference in fluorescence. Nevertheless, environmental conditions such as pH and dissolved oxygen also had negative effects on the fluorescence of GFP in *L. plantarum* [16].

### FaeLam is secreted by *L. plantarum* and *E. coli* without typical signal peptide

**Fig. 2:**
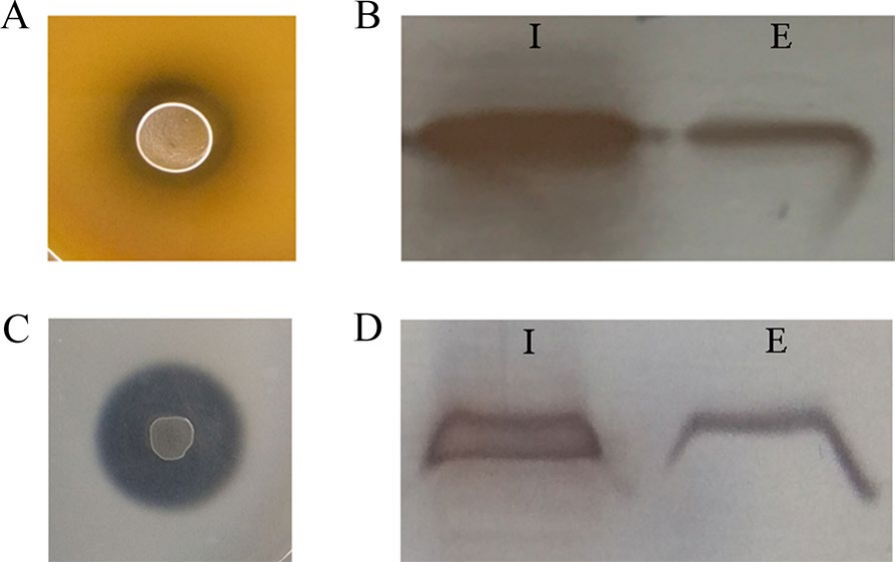
Activity assay and subcellular localization of FaeLam. **A** *L. plantarum* CGMCC6888 containing pLP-FaeLam grown in MRS medium supplementary with ethyl ferulate; **B** Western blot analysis of the subcellular localization of FaeLam expressed in *L. plantarum* CGMCC6888. **C** *E. coli* DH5α containing pLP-FaeLam grown in LB medium supplementary with ethyl ferulate; **D** Western blot analysis of the subcellular localization of FaeLam expressed in *E. coli* DH5α. I: intracellular fraction; E: extracellular fraction

We previously reported that *L. amylovorus* CGMCCC11056 was capable to produce feruloyl esterase FaeLam, which consists of 247 amino acids with a molecular mass of 27.4 kDa (accession number: AOR52353) [17]. Furthermore, no putative signal peptide motif was predicted at the N-terminal of FAE by using SignalP-5.0 server (http://www.cbs.dtu.dk/services/SignalP-5.0/) (Additional file 1: Figure S2A). The 744-bp of the *faeLam* gene was cloned from *L. amylovorus* CGMCC11056 and ligated into the P_tuf_-driven expression plasmid pLP3804, generating the pLP-FaeLam. This recombinant plasmid was transformed into *L. plantarum* CGMCC6888 and *E. coli* DH5α cells for overexpression. The recombinant cells could produce clear hydrolytic zone in the plates containing ethyl ferulate when incubation at 37 °C for 12 h, indicating that the FaeLam was correctly expressed (Fig. 2A, C). Furthermore, the distribution of FaeLam was investigated by detecting the activities of intracellular and extracellular fractions. As shown in Table 1, *E. coli* BL21(DE3) harboring pET22-FaeLam was used as a positive control. The extracellular pH drastically decreased when *L. plantarum* was cultured in MRS medium, resulting in a weak activity of FaeLam at acidic conditions. However, feruloyl esterase activity was detected in both intracellular and extracellular fractions of *L. plantarum* CGMCC6888 by HPLC (Additional file 1: Figure S3). Meanwhile, both of the fractions of *E. coli* DH5α containing plasmid pLP-FaeLam could produce hydrolytic zone according to the plate-based assay (Additional file 1: Figure S4).

**Table 1:** The intracellular and extracellular feruloyl esterase activities (U/mL) in different recombinant strains

Feruloyl esterase activities	*L. plantarum* CGMCC6888 With pLP3804-FaeLam	*E. coli* DH5α With pLP3804-FaeLam	*E. coli* BL21(DE3) With pET22b-FaeLam
Intracellular	397.1 ± 19.4	1742.3 ± 46.7	3886.4 ± 71.2
Extracellular	ND	540.9 ± 25.6	2013.7 ± 44.3

Feruloyl esterase activity was determined by using ρNPF as substrate. ND means no detectable activity via this method

The subcellular localization of FaeLam in *L. plantarum* CGMCC6888 and *E. coli* DH5α was further determined by western blot. As shown in Fig. 2B and D, FaeLam protein bands of the same molecular weight were observed in both of the intracellular and extracellular fraction. All these results suggested that *L. amylovorus* CGMCCC11056 FaeLam could be exported into the medium when expressed in *L. plantarum* CGMCC6888 and *E. coli* DH5α. Analysis of the N-terminal sequence of extracellular FaeLam of *E. coli* DH5α showed that it was MSRITIERDGL, which matched the amino acid sequence of FaeLam from residues 1 to 11 (Additional file 1: Figure S5). This result proved that FaeLam was translocated without removing any N-terminal sequence.

### Expression of inactive FaeLam

**Fig. 3:**
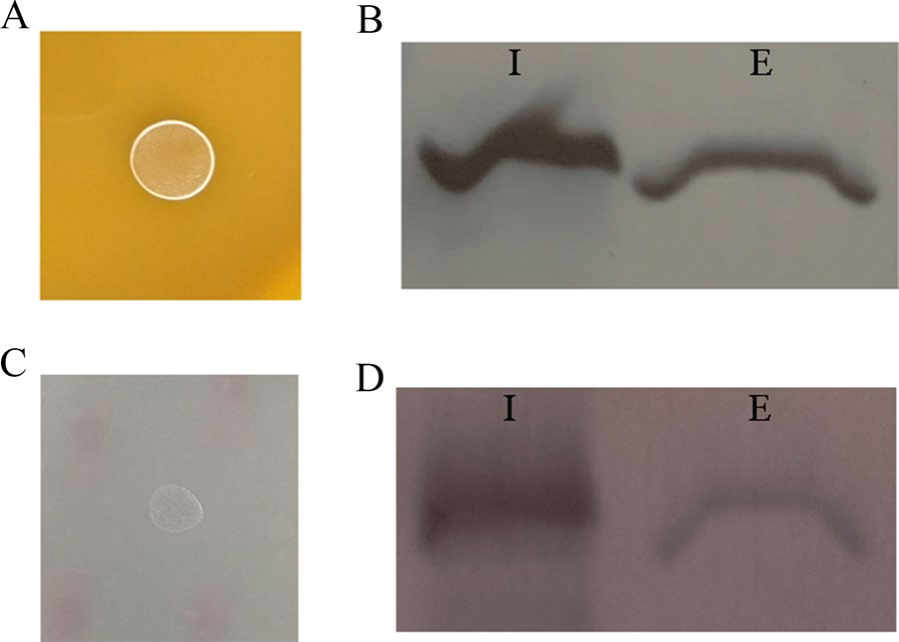
Activity assay and subcellular localization of FaeLam^S106A^. **A** *L. plantarum* CGMCC6888 containing pLP-FaeLam^S106A^ grown in MRS medium supplementary with ethyl ferulate; **B** Western blot analysis of the subcellular localization of FaeLam^S106A^ expressed in *L. plantarum* CGMCC6888; **C** *E. coli* DH5α containing pLP-FaeLam^S106A^ grown in LB medium supplementary with ethyl ferulate; **D** Western blot analysis of the **s**ubcellular localization of FaeLam^S106A^ expressed in *E. coli* DH5α. I: intracellular fraction; E: extracellular fraction

Su et al*.* [12] reported that *Thermobifida fusca* cutinase could be secreted from cells when heterologously expressed in *E. coli*, which resulted from its hydrolytic activity toward phospholipids. To exclude the possibility that FaeLam secretion was caused by its hydrolytic activity on the cell membrane, the active site of FaeLam was mutated according to the results of amino acid sequence alignment with other feruloyl esterases. *L. amylovorus* FaeLam belongs to the α/β-hydrolase family. The examination of FaeLam structure model reveals a Ser106-His225-Asp197 catalytic triad in which Ser106 is critical to the hydrolytic activity. Therefore, the site-directed mutagenesis of Ser106 to Ala was attempted. FaeLam^S106A^ was obtained and the plasmid pLP-FaeLam^S106A^ was constructed. When the mutant was expressed in *L. plantarum* CGMCC6888 and *E. coli* DH5α, the feruloyl esterase activity of recombinant cells was not detected (Fig. 3A, C). However, the FaeLam^S106A^ protein band was still detected in both of the intracellular and extracellular fractions of *L. plantarum* CGMCC6888 and *E. coli* DH5α (Fig. 3B, D).

### Expression of FaeLam with N-terminal deletion

**Fig. 4:**
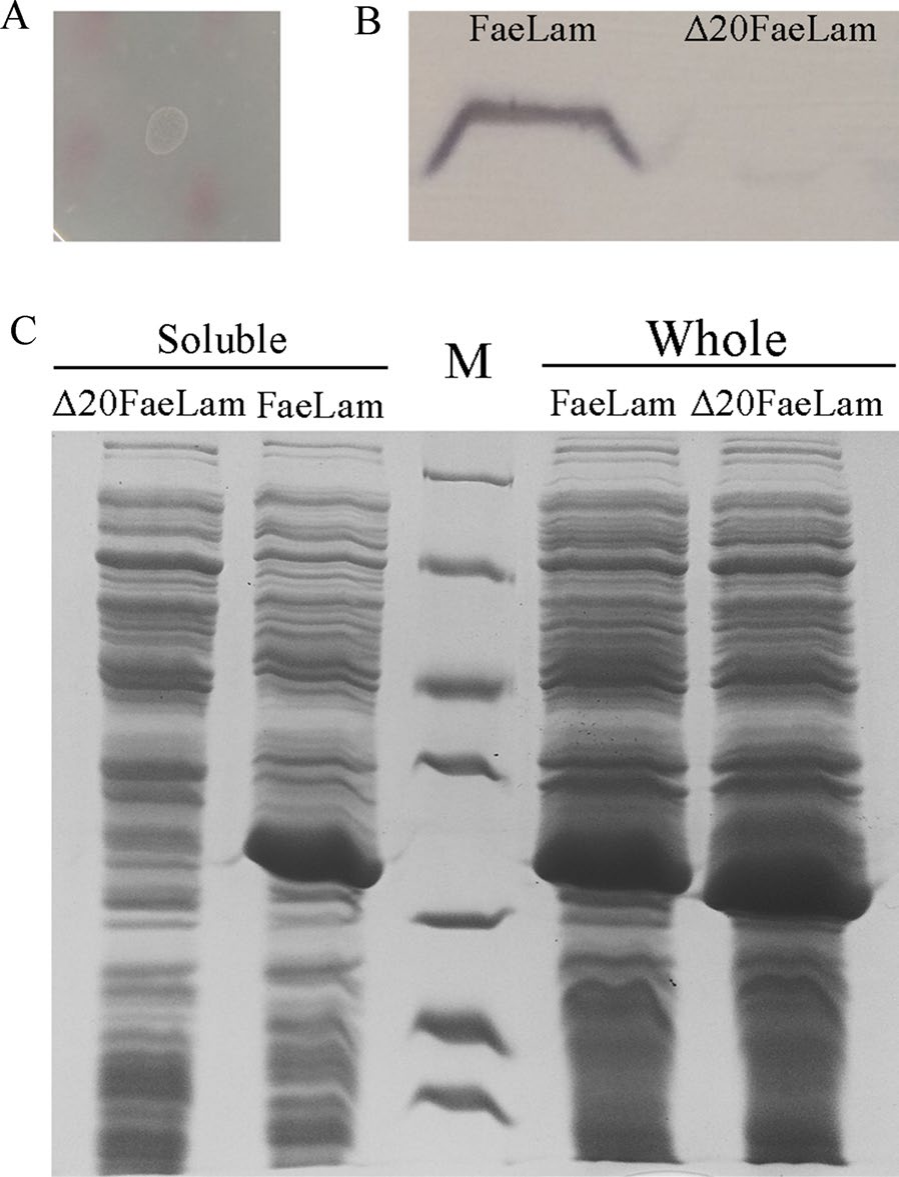
Activity assay and subcellular localization of Δ20FaeLam. **A** *E. coli* DH5α containing pLP-Δ20FaeLam grown in LB medium supplementary with ethyl ferulate. **B** Western blot analysis of the extracellular proteins of *E. coli* DH5α containing pLP-FaeLam and pLP-Δ20FaeLam. **C** SDS-PAGE analysis of the whole cell protein and soluble cell extracts of *E. coli* DH5α containing pLP3804-FaeLam and pLP3804-Δ20FaeLam. M: Marker (from top to bottom: 116, 66.2, 45, 35, 25, 18.4, 14.4 kDa)

Since the secretion of FaeLam was not due to the hydrolysis activity, whether the N-terminus of FaeLam played a role in its secretion was further investigated. The FaeLam protein mutant (Δ20FaeLam) with 20 amino acid truncated at the N-terminus was constructed. When *E. coli* DH5α was transformed with pLP-Δ20FaeLam, the recombinant cells could not produce hydrolytic zone in the plate (Fig. 4A), and the mutant protein was not detected in extracellular fraction by western blot (Fig. 4B). The same phenomenon was observed in *L. plantarum* CGMCC6888 harboring the plasmid pLP-Δ20FaeLam (data not shown). Furthermore, the SDS-PAGE was performed to analysis the expression of FaeLam and Δ20FaeLam in *E. coli* DH5α. As shown in Fig. 4C, these two proteins were expressed with almost no difference as revealed in the whole cells analysis. However, the Δ20FaeLam was not detected in the soluble fraction, suggesting it was deposited as inclusion bodies. Taken together, deletion of N-terminus completely inhibited the formation of the soluble enzyme, indicating the N-terminal region of FaeLam might play an important role in its secretion like the Cel-CD [13].

### Secretion of heterologous protein by the N-terminus

**Fig. 5:**
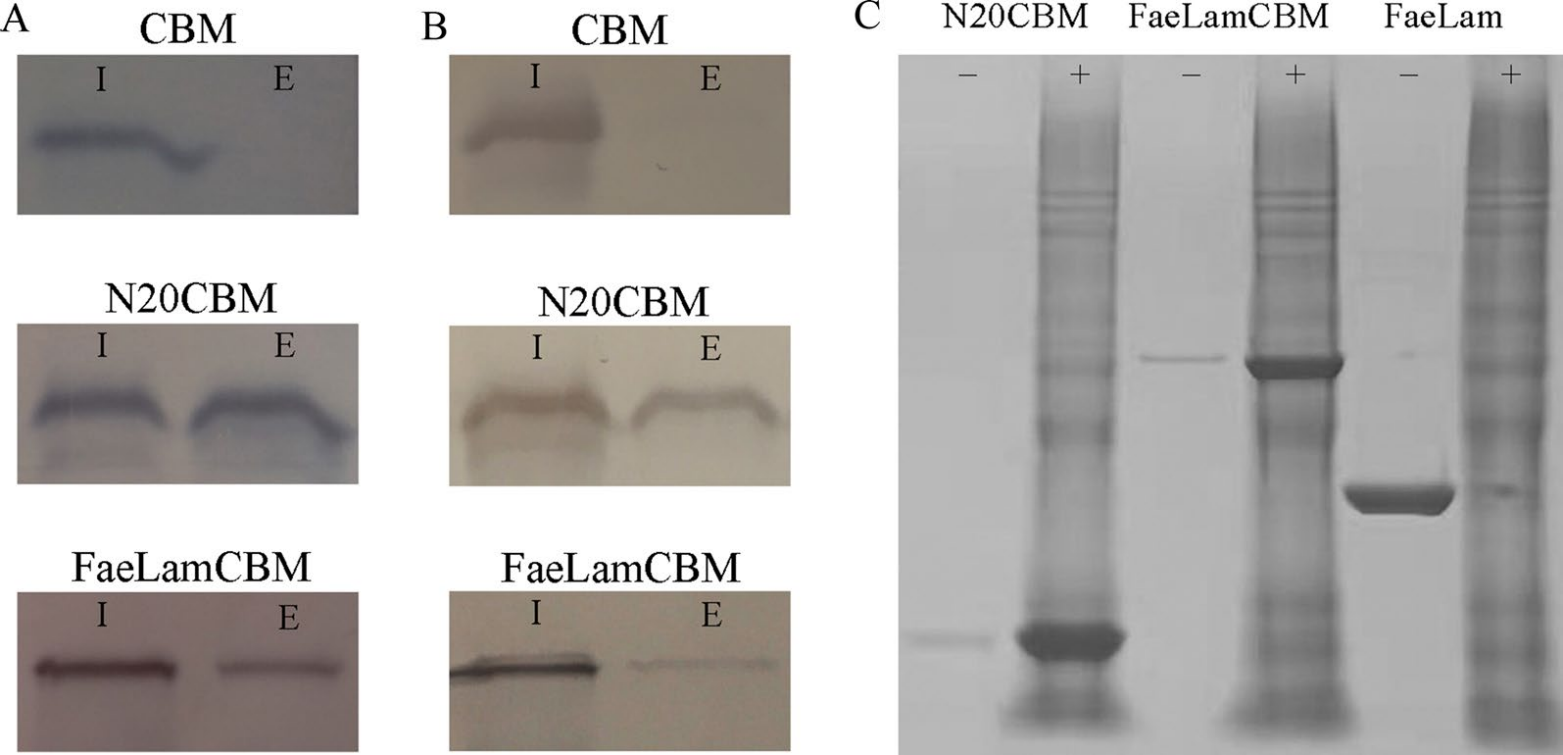
The translocation and activity of heterologous protein fused with FaeLam or its N-terminus. **A** Western blot analysis of the subcellular localization of CBM, N20CBM and FaeLamCBM expressed in *L. plantarum* CGMCC6888; **B** Western blot analysis of the subcellular localization of CBM, N20CBM and FaeLamCBM expressed in *E. coli* DH5α; **C** SDS-PAGE analysis of the binding abilities of N20CBM, FaeLamCBM and FaeLam to de-starched wheat bran. I: intracellular fraction; E: extracellular fraction; −: unbound fraction; +: fraction bound to de-starched wheat bran

To directly verify the N-terminus sequence plays the key role in secretion, the N-terminal 20 amino acid (N20) was used to guide the heterologous protein out of *L. plantarum* and *E. coli*. The carbohydrate binding module of xylanase (CBM, 14.2 kDa) from *Paenibacillus panacisoli* was selected and fused downstream to the N20, resulting of N20CBM [18]. As shown in the Fig. 5A and B, this fusion protein were detected in the extracellular after 12 h cultivation of *L. plantarum* CGMCC6888 and *E. coli* DH5α. Furthermore, we also tested if the FaeLam could be used as a fusion partner to carry CBM out of the cells in recombinant *L. plantarum* and *E. coli*. The results showed that FaeLamCBM could also be secreted into the medium after 12 h cultivation. As the controls, the CBM can only be detected in the intracellular fraction when it was expressed without any additional sequences. This result also indicated that the detected extracellular proteins were not due to cell lysis. Therefore, the N20 could act as a signal peptide.

The N20CBM and FaeLamCBM were purified from the extracellular fraction of *E. coli*. The function of the CBM in the fusion proteins was identified by the binding abilities to the de-starched wheat bran. As shown in Fig. 5C, N20CBM and FaeLamCBM decreased from the unbound fraction, while prominent bands were detected in the bound fraction. Meanwhile, FaeLam which was used as a control still existed in the unbound fraction. These results indicated that CBM contained in the two fusion proteins had the ability to absorb the corresponding carbohydrate.

## Discussion

Polysaccharide-bound hydroxycinnamic acids are widely present in cell walls [19]. Feruloyl esterases can cleave the ester bond between hydroxycinnamic acids and polysaccharide. The most reported feruloyl esterases are natural extracellular enzymes in their native organisms, which is beneficial for the enzymes to access their substrates [20]. However, it is still controversial that feruloyl esterases derived from *Lactobacillus* are located in intracellular or secreted out of cells. Feruloyl esterase has been identified in many *Lactobacillus* species, including *L. gasseri* [21], *L. acidophilus* [22], *L. reuteri*, *L. casei* [23], *L. helveticus* [24], *L. johnsonii* [7], *L. fermentum* [6] and *L. crispatus* [25]. Although bioinformatic analysis showed that no signal peptides were predicted in these *Lactobacillus* feruloyl esterases, the increase of free phenolic acids was detected in whole grain barley and oat groat by fermentation with *Lactobacillus* strains, indicating the secretory characteristics of their feruloyl estreases [23, 26]. Our results could shed light on the location of feruloyl esterases produced by *Lactobacillus* species. Furthermore, the *L. plantarum* with secreted feruloyl esterase might play roles in many applications. For example, it could be applied in ensiling of crop for improving fermentation quality and fibre digestibility of ensiled forages, and could be orally administered as a dietary supplement or functional food for increasing the intestinal feruloyl esterase activity to enhance the bioavailability of hydroxycinnamic acids, thus improving oxidative status [27, 28].

Our previous experiments showed that FaeLam could be secreted from strain *E. coli* BL21(DE3) [11]. In the present study, it could also be secreted into extracellular environment of *E. coli* DH5α, suggesting the secretion of FaeLam is not strain dependent. Since feruloyl esterase has significant potential applications in many industrial fields, the preparation of feruloyl esterase has drawn extensive attention in recent years. Therefore, these *E. coli* strains expressing the *Lactobacillus* feruloyl esterases are ideal hosts for feruloyl esterases production, although optimization is needed to improve the yields and productivity in the future. The secretion characteristic allows a simple process of product separation and purification. Furthermore, the recombinant *E. coli*, as well as the recombinant *L. plantarum*, can be directly used to produce value-added products from agricultural wastes, such as ferulic acid and vanillin [29, 30].

It was proven that the N-terminal sequence of FaeLam played a crucial role in this secretion. Bioinformatic analysis revealed that the N-terminus of FaeLam had good polarity and hydrophilicity (Additional file 1: Figure S2B, C). These characteristics were significantly different from the Sec signal peptide [31]. Thus, the feruloyl esterase could be classified as non-classically secreted protein, which seldom reported in lactic acid bacteria [32]. In our previous study, we found that ten feruloyl esterases produced by different lactic acid bacteria all could be secreted by *E. coli*, although with diverse secretion levels [11]. Sequence alignment showed that the N20 of these enzymes had a certain similarity, especially conserved in two leucine and a glycine (Additional file 1: Figure S6). Designing mutated enzymes at these sites will help to understand the role of different residues in secretion. In addition, although the sequence basis of the feruloyl esterase for secretion was determined in this work, it is necessary to further confirm whether there are related proteins required for the feruloyl esterase transportation in the strains, thus to fully understand the non-classical secretion pathway of feruloyl esterase [33].

We also demonstrated that FaeLam and its N-terminal 20 amino acid residues can be used as carriers for extracellular production of heterologous target proteins in *L. plantarum* CGMCC6888 and *E. coli* DH5α. To our best knowledges, this is the first report to show that a short peptide can serve as a signal peptide and guide heterologous protein out of the cells of both Gram-positive and Gram-negative bacteria. During the past years, numerous efforts have been made to explore the potential of *L. plantarum* and *E.coli* as cell factories for secreted expression recombinant proteins [34, 35]. As for *L. plantarum*, heterologous protein secretion dependent on typical Sec pathway is usually very inefficient, and cytoplasmic proteins are usually not translocated across the cell membrane with the aid of signal peptides [36, 37]. These factors limit the use of *L. plantarum* as a cell factory and highlight the importance of exploring new transport routes for secreted heterologous proteins. *E. coli*, a Gram-negative bacterium, has two membranes, which makes it even harder to secrete heterologous proteins than in Gram-positive bacterium. Secretory production of proteins in *E. coli* has many possible applications, and will simplify the protein production process and decrease production costs of enzymes [38, 39]. Therefore, the N-terminus of FaeLam provides a new tool for the secretion of recombinant proteins in *L. plantarum* and *E. coli*.

## Conclusions

The feruloyl esterase derived from *L. amylovorus* could be secreted to the extracellular of *L. plantarum* CGMCC6888 and *E. coli* DH5α. We proved the N-terminus of FaeLam played the important role in its secretion. Although further studies should be conducted for fully understanding the secretion mechanism, the present study provided alternative strains for the production of feruloyl esterase and ferulic acid. In addition, the N-terminus of FaeLam also could be employed as carriers for extracellular production of recombinant proteins. *L. plantarum* and *E. coli* are important model species in molecular biology research and often used as expression hosts for heterologous proteins. Therefore, a new method was established for protein secretion in *L. plantarum* and *E. coli* through fusion the targeted protein to N20 of FaeLam.

## Material and methods

### Bacterial strains, culture conditions, plasmids and chemicals

The strains *L. amylovorus* CGMCC11056 and *L. plantarum* CGMCC6888 were cultured statically at 37 °C in MRS (de Man, Rogosa and Sharpe) broth (Oxoid, Basingstoke, UK). *E. coli* DH5α was cultured in LB (Luria–Bertani) medium at 37 °C aerobically. If necessary, chloramphenicol was added with a final concentration of 10 μg/mL for *E. coli* or 5 μg/mL for *L. plantarum*. The plasmids used in this study were listed in Table 2. Methyl ferulate and ethyl ferulate used as substrates for enzyme assays of feruloyl esterase, were purchased from Sigma Chemicals Industries Co., Ltd. (SanFrancisco, USA). All PCR reactions were performed with Ex Taq polymerase (TaKaRa, Tokyo, Japan). Restriction enzymes and T_4_ DNA ligase were also purchased from TaKaRa Biotechnology Co., Ltd. (Tokyo, Japan). DNA extraction kit, agarose gel DNA purification kit, and cycle pure kit were obtained from Omega Bio-tek (Atlanta, USA).

**Table 2:** The plasmids used in this study

Plasmids	Description	Source
pUC19	Amp^r^, ori	Novagen
pD403	2.8 kb cryptic plasmid of *L. planturum* D403	Our laboratory
pET22b-FaeLam	Amp^r^, pET-22b vector ligated with *faeLam* gene	Our laboratory
pNZ8148	Cm^r^	Mierau and Kleerebezem [41]
pMN402	Derivation of *gfp* gene	Scholz et al. [15]
pLP3804	Cm^r^, ori from pUC19, ori and repA from pD403, promoter P_tuf_	This study
pLP-GFP	pLP3804, *gfp* gene under promoter P_tuf_	This study
pLP-FaeLam	pLP3804, *faeLam* gene under promoter P_tuf_	This study
pLP-Δ20FaeLam	pLP3804, *faeLam* gene that truncated 60 bp at 5′region	This study
pLP-FaeLam^S106A^	pLP3804, the mutated *faeLam* gene under promoter P_tuf_	This study
pLP-CBM	pLP3804, *cbm* from *Paenibacillus panacisoli* under promoter P_tuf_	This study
pLP-N20CBM	pLP3804, *n20* gene fusion with *cbm* from *Paenibacillus panacisoli*	This study
pLP-FaeLamCBM	pLP3804, *faeLam* gene fusion with *cbm* from *Paenibacillus panacisoli*	This study

### DNA manipulations

To express proteins in both of *L. plantarum* and *E. coli*, a shuttle vector pLP3804 was constructed. The primers used in this study were listed in Additional file 1: Table S1. The promoter (P_tuf_) of the putative elongation factor and the terminator (Ter) were amplified with the genomic DNA of *L. plantarum* CGMCC6888 and *Lactococcus lactic* MG1363 as templates, respectively [14]. The *repA* of pD403 and the replicon of pUC19 were responsible for replication of the vector in *L. plantarum* and *E. coli* [40]. The chloramphenicol resistance gene derived from pNZ8148 was used as the selection marker [41]. The multiple cloning sites were included in the synthetic primers.

The *faeLam* gene encoding FaeLam was amplified from *L. amylovorus* CGMCC11056 using the primers FaeLam-F and FaeLam-R-His_6_ (Table 3) [17]. Then the purified PCR product was digested with the restriction enzymes *Nde*I and *Xho*I and ligated into the expression vector pLP3804 restricted with the same enzymes, obtaining the recombinant plasmid pLP-FaeLam. The mutant plasmid pLP-FaeLam^S106A^ was generated by utilizing the overlap extending methodology with the primers S106A-F and S106A-R, and confirmed by hydrolytic activity determination. The DNA sequence encoding FaeLam with 20 amino acid residues deletion at the N-terminus was amplified from the plasmids pLP-FaeLam with primers FaeLamΔ20-F and FaeLam-R-His_6_, generating the plasmid pLP-Δ20FaeLam.

**Table 3:** The primers used in this study

Primer	Sequence(5′-3′)	Restriction sites
GFP-F	GCGCATATGAGCAAAGGAGAAGAAC	*Nde*I
GFP-R	ATAGGATCCTTAGTATAGCTCATCCATG	*BamH*I
FaeLam-F	TATACATATGTCCCGCATTACAATTG	*Nde*I
FaeLam-R-His_6_	GCGCTCGAGTTAGTGGTGGTGGTGGTGGTGGAATAATGGTTTTAAAAATT	*Xho*I
S106A-F	GTCATGCTCAAGGCGGCG	
S106A-R	CGCCGCCTTGAGCATGACCTACTAAAAAGATATTGC	
FaeLamΔ20-F	ATACATATGTTTGGCGAAATTTATGACATG	*Nde*I
CBM-F1	ACACTTACGATTGGAGGCAG	
CBM-F2	ATACATATGACACTTACGATTGGAGGCAG	*Nde*I
CBM-R-His_6_	ATACTCGAGTTAGTGATGATGATGATGATGATGGATGTCCAAATAGTC	*Xho*I
N20CBM-R	GCCTCCAATCGTAAGTGTAGGCTCTTCACGATCTCC	
FaeLamCBM-R	GCCTCCAATCGTAAGTGTCTAGAATAATGGTTTT	

To fuse the protein CBM derived from *P. panacisoli* to the N20 amino acid residues of FaeLam [18], primer sets FaeLam-F/N20CBM-R and CBM-F1/CBM-R-His_6_ were used to amplify the N20 and CBM sequences, and then these two fragments were fused by the overlap extending methodology. The resulting product was digested with the restriction enzymes *Nde*I and *Xho*I and ligated into the corresponding sites of vector pLP3804 to obtain the plasmid pLP-N20CBM. The same method was used to fuse genes *faeLam* and *cbm*, and recombinant plasmid pLP-FaeLamCBM was obtained. Furthermore, pLP-CBM was constructed as a control by using primers CBM-F2 and CBM-R-His_6_. All the resulting plasmids were confirmed by DNA sequencing.

### Transformation

To prepare the *L. plantarum* competent cells, an overnight culture was transferred into 5 mL of SGMRS (MRS added 0.75 M sorbitol and 1% glycine) as the inoculation volume of 1%, and incubated at 37 °C. When the OD_600_ reached 0.6, cultures were centrifuged at 4000×*g*, 4 °C for 5 min. The sedimented cells were washed with electroporation buffer (0.05 M sucrose, 1 mM MgCl_2_) three times and then resuspended in 40 μL electroporation buffer. For electro-transformation of *L. plantarum*, the 40 μL fresh prepared competent cells were gently mixed with 500 ng DNA and left on ice for 5 min. The mixture was transferred into a pre-cooled cuvette with a gap of 0.2 cm (Bio-Rad, California, USA) and electroporated by a pulse of 2000 V at 25 μF (Gene Pulser, Bio-Rad, California, USA). After electroporation, 960 μL of SMRS broth (MRS with 0.75 M Sorbitol and 10 mM CaCl_2_) was immediately added and the cells were incubated at 37 °C for 2 h. Competent cells of *E. coli* DH5α were prepared and transformed with plasmid DNA as described in the previous study [42]. To select the transformants, the culture was plated on LB or MRS plates containing chloramphenicol with appropriate concentrations.

### Fluorescence assay

Recombinant strains harboring the pLP3804 and pLP-GFP were cultured and taken out after 12 h for the fluorescence measurement. The cells were collected by centrifugation at 10,000×*g* for 5 min, and then washed twice using PBS buffer (137 mM NaCl, 2.7 mM KCl, 10 mM Na_2_HPO_4_, 2 mM KH_2_PO_4_, pH 7.4). After being resuspend in an equal volume of buffer, 200 μL of bacterial suspension was transferred into a 96-well plate. The fluorescence was detected by a Multi-Detection Microplate Reader with the excitation at 485 nm and the emission at 528 nm.

### Feruloyl esterase activity assay

The plate-based assay to preliminarily determine feruloyl esterase activity was as follows. Ethyl ferulate with a concentration of 6.7 mM was added in the MRS and LB plates as substrate. Strains or samples were loaded into the plate, and then incubated at 37 °C. The hydrolytic zone was examined and photographed. Furthermore, the ρNPF was used as substrate to quantitatively determine the feruloyl esterase activity according to our previous work [11].

High performance liquid chromatography (HPLC) method was also employed to detect the hydrolytic activity of feruloyl esterase. The enzyme reaction was carried out in 1 mL of intracellular or extracellular fraction containing 1 mM methyl ferulate. After 12 h incubation at 37 °C, 1 mL 50% acetate (v/v) was added to terminate the reaction. The HPLC was equipped with an XBridge BEH300 C18 reverse phase column (150 mm × 4.6 mm; Waters, Milford, USA) at a flow rate of 1.0 mL/min. The mobile phase was composed of solvent A (methanol) and solvent B (water and acetic acid, 99:1, v/v) at a ratio of 1:1, and the column eluent was monitored at the A_320_ with temperature of 35 °C [17].

### Functional analysis of the carbohydrate binding module

To verify the function of CBM contained in the fusion proteins, their binding ability to de-starched wheat bran was detected. The cultures of *E. coli* secreting N20CBM and FaeLamCBM were centrifugated at 10,000×*g*, 4 °C for 10 min. The supernatant was collected and filtered by 0.22-μm filter. Extracellular proteins were concentrated using ultrafiltration tubes (Millipore, Massachusetts, USA) and dialyzed against PBS buffer. Then, the target proteins were obtained by the affinity chromatography of Ni-TED column (GE Healthcare, Stockholm, Sweden). Subsequently, the purified proteins were mixed with de-starched wheat bran at 5% (w/v) final concentration and kept at 4 °C for 3 h. After centrifuged at 10,000 × g, 4 °C for 10 min, the supernatant was collected as unbound fraction. The pellets were washed with PBS buffer, and then the bound fraction was eluted by washing with 1 × SDS-PAGE loading buffer.

### SDS-PAGE and western blot analysis

Sodium dodecyl sulfate-polyacrylamide gel electrophoresis (SDS-PAGE) with a 5% stacking gel and a 12% separating gel was used to separate the protein bands, and then protein bands in the gel were visualized by Coomassie brilliant blue staining.

To conduct western blot analysis, a PVDF membrane was used to electro-transfer the separated proteins in SDS-PAGE gel at 200 mA with appropriate time, and then blocked with 5% non-fat milk for 1 h. Considering that the C-terminus of target proteins was labeled a 6 × His tag, the primary antibody anti-six-histidine mouse IgG (1:1,000 dilution; Novagen, USA) was used to incubate the membrane for 12 h. Subsequently, the membrane was washed three times with TBST buffer (20 mM Tris–HCl, 500 mM NaCl, 0.01% Tween-20, pH 7.4). The secondary antibody horseradish-peroxidase (HRP)-labeled goat anti-mouse IgG (1:300 dilution; Solarbio, China) was used to incubate the membrane for 1 h. The positive protein bands in the PVDF membrane were visualized using a HRP-DAB kit (Tiangen, Beijing, China).

### Cell fractionation

For enzymatic analysis, intracellular and extracellular were prepared as follows. The culture broth after 12 h cultivation was centrifuged at 10,000×*g*, 4 °C for 10 min, and the supernatant was collected and filtered by 0.22-μm filter as the extracellular fraction. The centrifuged cells were washed and resuspended in an equal volume of PBS buffer, and then disrupted by a Precellys 24 (Bertin, Paris, France). After centrifugation at 10,000×*g*, 4 °C for 10 min, the supernatant was collected and filtered as intracellular fraction.

For western blot analysis, cell fractionation of *L. plantarum* and *E. coli* was performed as follows. Five milliliter of *L. plantarum* CGMCC6888 culture broth after 12 h cultivation was centrifuged at 10,000×*g*, 4 °C for 10 min, the resulting supernatant was filtered through a 0.22-μm filter and ice-cold trichloroacetic acid was added as the final concentration of 10% (v/v). After incubation on ice for 1 h, the supernatant was centrifuged at 12,000×*g*, 4 °C for 15 min, and the precipitate was washed twice with ice-cold acetone, and then it was air-dried and dissolved in 100 μL PBS buffer to obtain the extracellular fraction. The centrifuged cells were washed and resuspended in 1 mL PBS buffer, and then disrupted by a Precellys 24 (Bertin, Paris, France). After centrifugation at 10,000×*g*, 4 °C for 10 min, the supernatant was collected as intracellular fraction. One milliliter of *E. coli* DH5α culture broth after 12 h cultivation was centrifuged at 10,000×*g*, 4 °C for 10 min, and the supernatant was collected and filtered by 0.22-μm filter as the extracellular fraction. The preparation of *E. coli* DH5α intracellular fraction was same as that of *L. plantarum* CGMCC6888.

## Supplementary Information

**Additional file 1: Figure S1.** SDS-PAGE analysis of the expression of green fluorescence protein in *L. plantarum* CGMCC6888 and *E. coli* DH5α. **Figure S2.** The signal peptide prediction of FaeLam by using SignalP-5.0 server (A). The polarity (B) and hydrophilicity (C) prediction of FaeLam by using Protscale. **Figure S3.** HPLC analysis of methyl ferulate degradation by intracellular (A) or extracellular (B) fractions of *L. plantarum* CGMCC6888 harboring pLP-FaeLam. The corresponding heat-inactivated intracellular (C) or extracellular (D) fractions were also conducted as controls. The peaks for ferulic acid and methyl ferulate were detected at 2.5 min and 4 min, respectively. **Figure S4.** Feruloyl esterase activity analysis of intracellular (A) or extracellular (B) fractions of *E. coli* DH5α harboring pLP-FaeLam. **Figure S5.** Analysis of the N-terminal sequence of extracellular FaeLam. **Figure S6.** Sequence alignment of feruloyl esterases from different *Lactobacillus* strains by using Clustal Omega. The 20 amino acid of N-terminus was circled. **Figure S7.** The full gels of the western blot results. Targeted bands were circled by red square. **Table S1.** Primers used in the vector construction of pLP3804.

## Data Availability

All data generated or analysed during this study are included in this published article.

## Abbreviations

N20: 20 Amino acids of N-terminus
GRAS: Generally recognized as safe
GFP: Green fluorescence protein
CBM: Carbohydrate binding module
IPTG: Isopropyl-β-d thiogalactopyranoside
SDS-PAGE: Sodium dodecylsulphate polyacrylamide gel electrophoresis
HPLC: High performance liquid chromatography
