# Supplementary material for: The N-terminus of Lactobacillus amylovorus feruloyl esterase plays an important role in its secretion by Lactobacillus plantarum and Escherichia coli
Source: Microb Cell Fact. 2021 Aug 3;20:152. doi: 10.1186/s12934-021-01645-9 (PMC8335865; doi:10.1186/s12934-021-01645-9)
Supplement: Supplementary file 1 — Additional file 1: Figure S1. SDS-PAGE analysis of the expression of green fluorescence protein in L. plantarum CGMCC6888 and E. coli DH5α. Figure S2. The signal peptide prediction of FaeLam by using SignalP-5.0 server (A). The polarity (B) and hydrophilicity (C) prediction of FaeLam by using Protscale. Figure S3. HPLC analysis of methyl ferulate degradation by intracellular (A) or extracellular (B) fractions of L. plantarum CGMCC6888 harboring pLP-FaeLam. The corresponding heat-inactivated intracellular (C) or extracellular (D) fractions were also conducted as controls. The peaks for ferulic acid and methyl ferulate were detected at 2.5 min and 4 min, respectively. Figure S4. Feruloyl esterase activity analysis of intracellular (A) or extracellular (B) fractions of E. coli DH5α harboring pLP-FaeLam. Figure S5. Analysis of the N-terminal sequence of extracellular FaeLam. Figure S6. Sequence alignment of feruloyl esterases from different Lactobacillus strains by using Clustal Omega. The 20 amino acid of N-terminus was circled. Figure S7. The full gels of the western blot results. Targeted bands were circled by red square. Table S1. Primers used in the vector construction of pLP3804. [file 12934_2021_1645_MOESM1_ESM.docx]

**Figure S1:** SDS-PAGE analysis of the expression of green fluorescence protein in *L. plantarum* CGMCC6888 and *E. coli* DH5α.

**
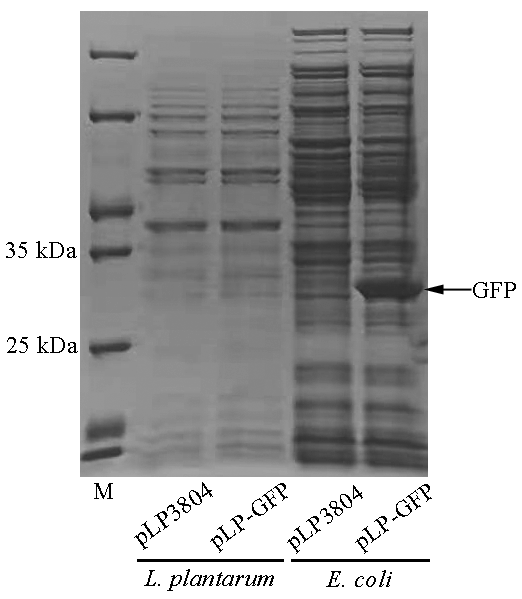
**

**Figure S2:** The signal peptide prediction of FaeLam by using SignalP-5.0 server (A). The polarity (B) and hydrophilicity (C) prediction of FaeLam by using Protscale.

**
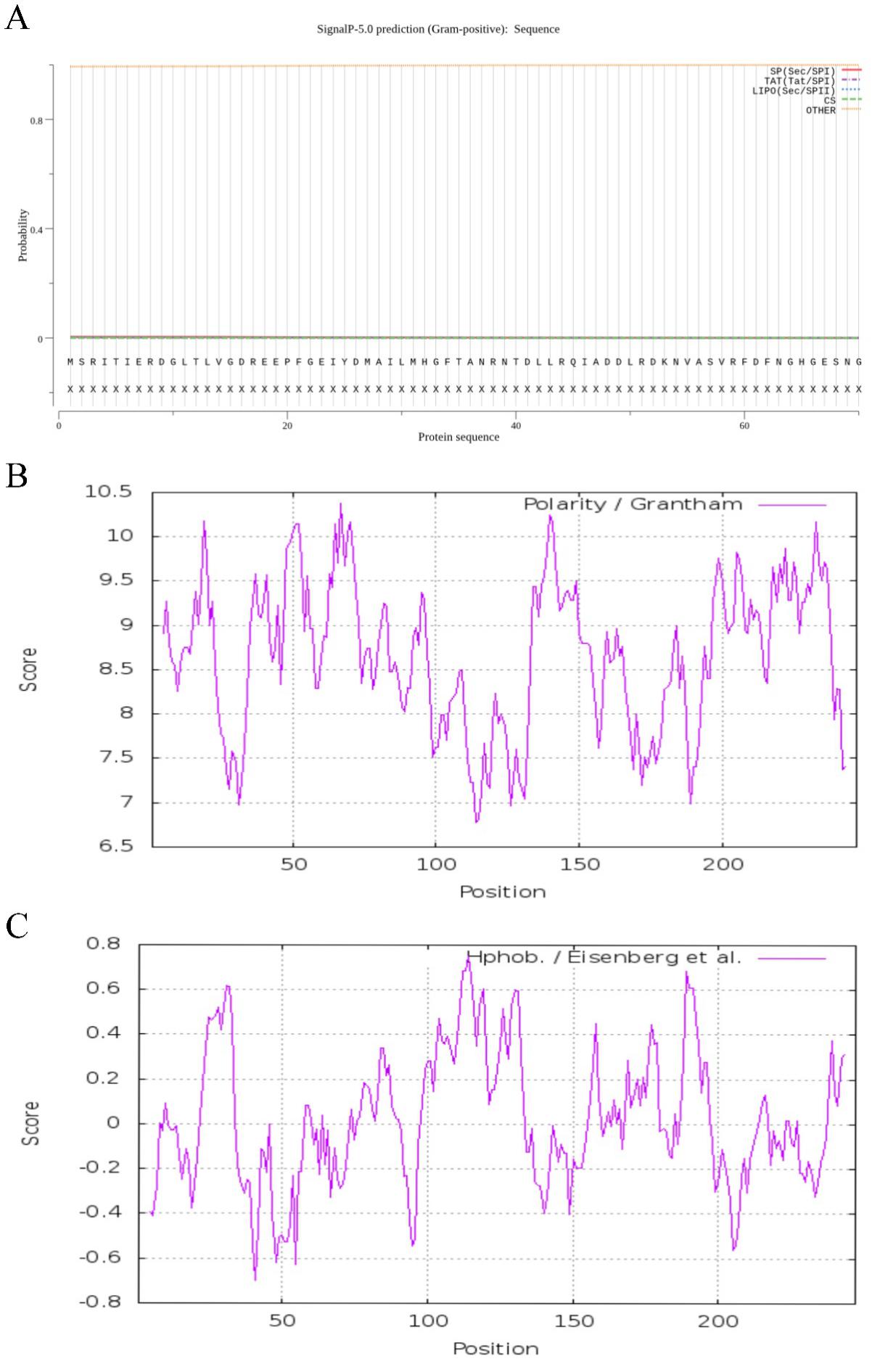
**

**Figure S3:** HPLC analysis of methyl ferulate degradation by intracellular (A) or extracellular (B) fractions of *L. plantarum* CGMCC6888 harboring pLP-FaeLam. The corresponding heat-inactivated intracellular (C) or extracellular (D) fractions were also conducted as controls. The peaks for ferulic acid and methyl ferulate were detected at 2.5 min and 4 min, respectively.


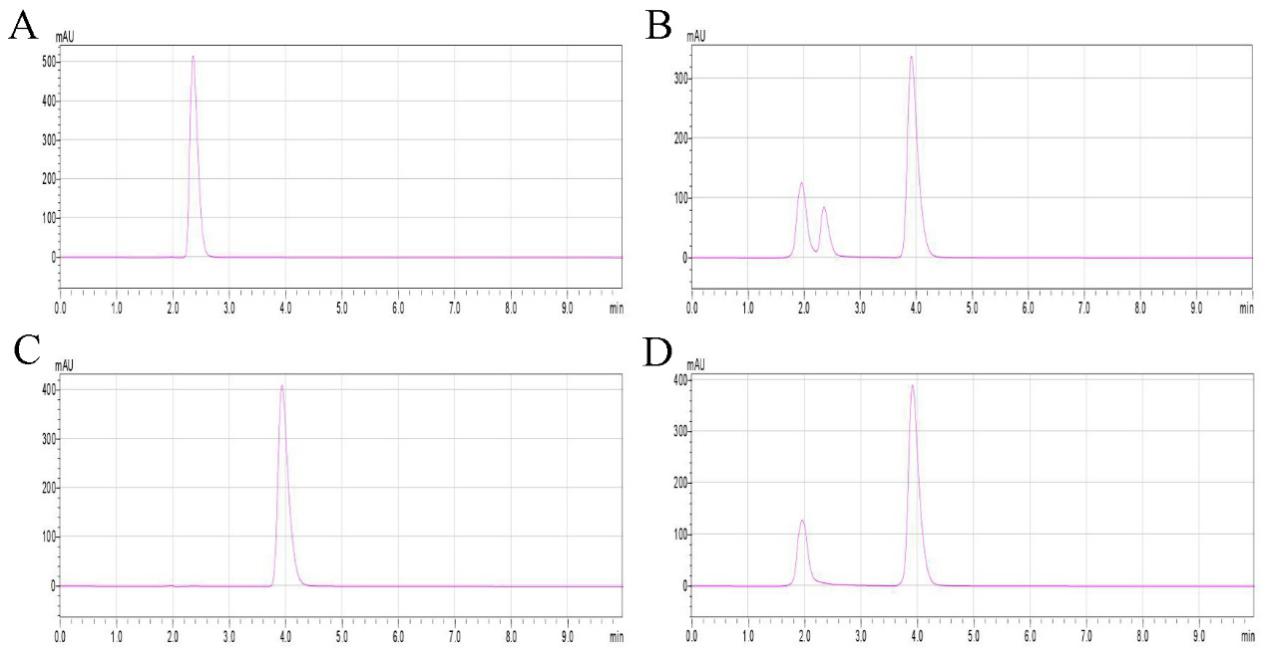


**Figure S4:** Feruloyl esterase activity analysis of intracellular (A) or extracellular (B) fractions of *E. coli* DH5α harboring pLP-FaeLam.

**
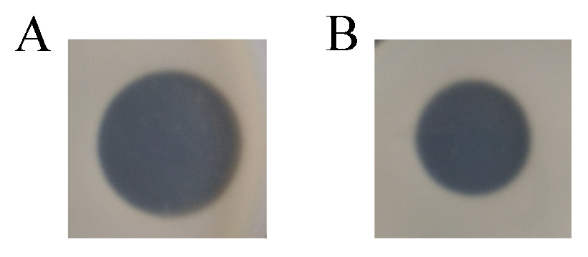
**

**Figure S5:** Analysis of the N-terminal sequence of extracellular FaeLam


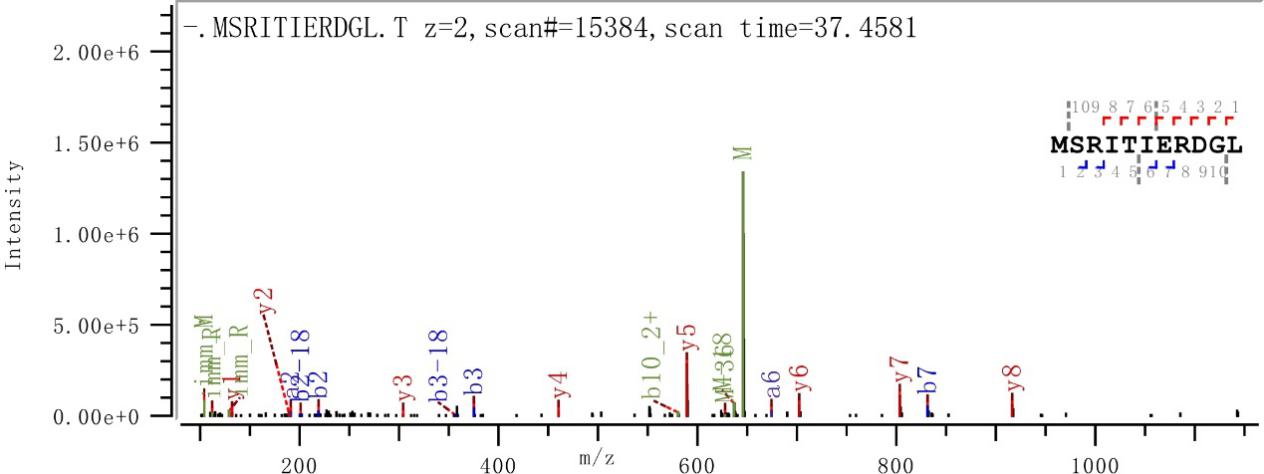


**Figure S6:** Sequence alignment of feruloyl esterases from different *Lactobacillus* strains by using Clustal Omega. The 20 amino acid of N-terminus was circled.


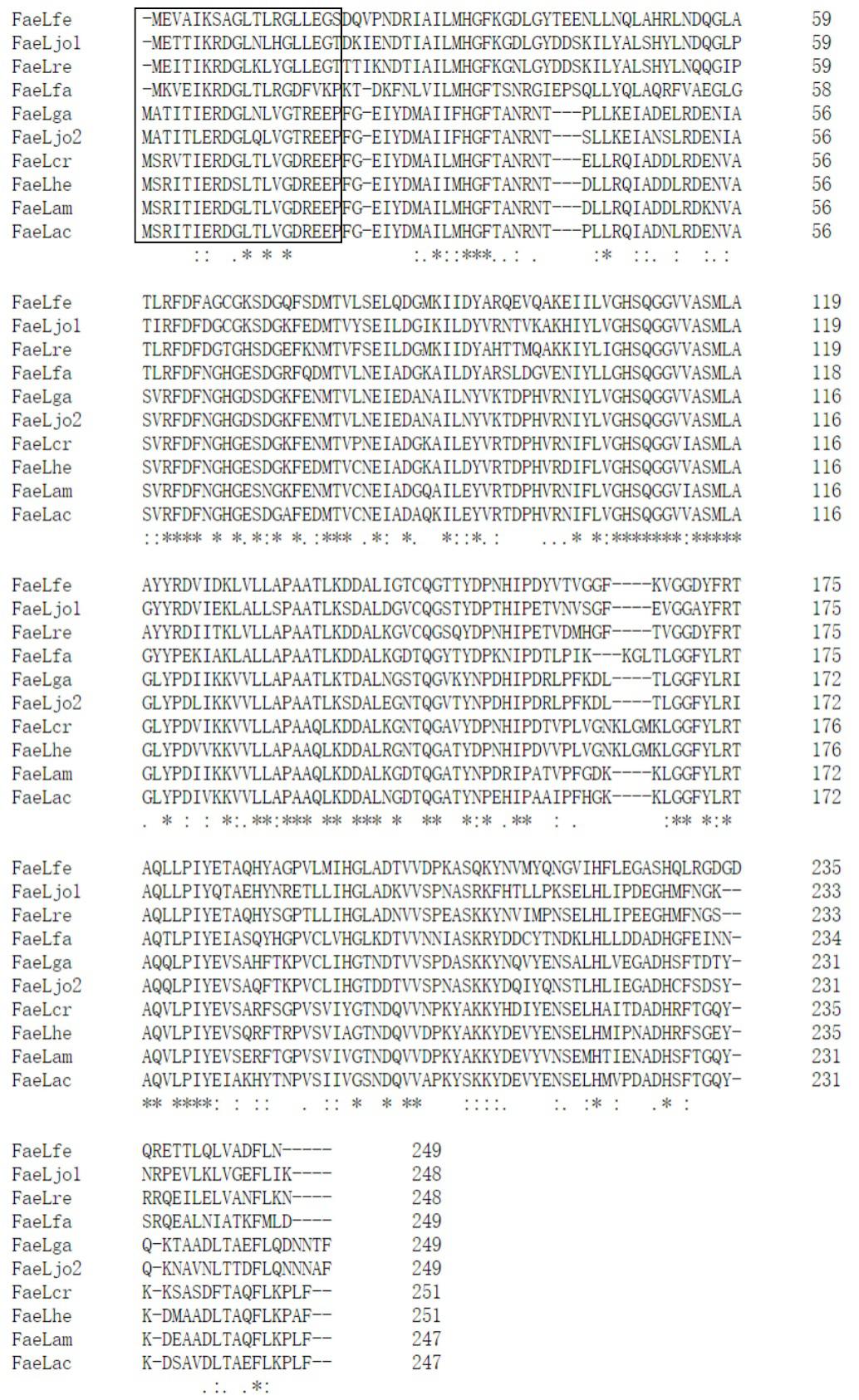


**Figure S7:** The full gels of the western blot results. Targeted bands were circled by red square.


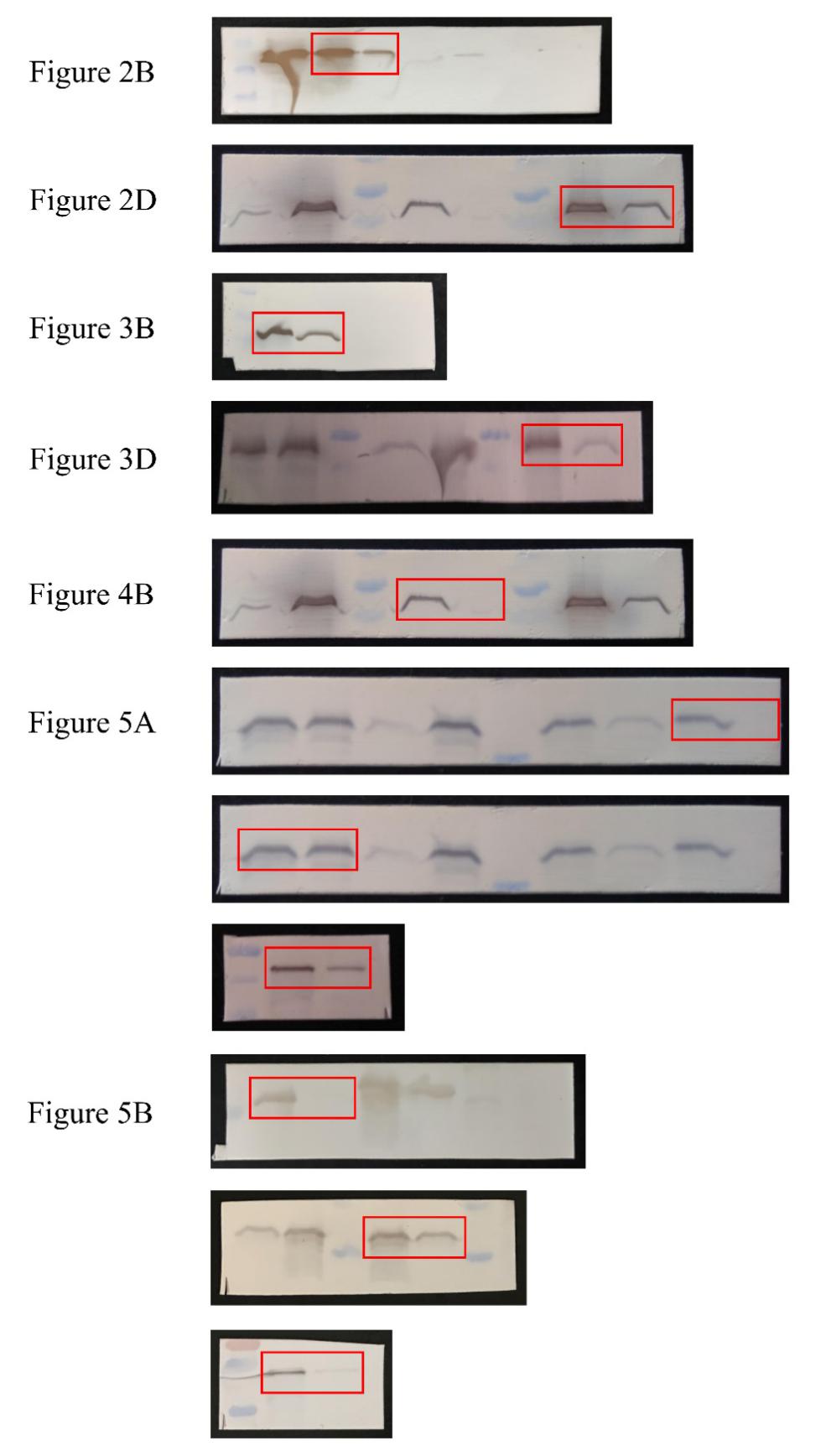


Table S1: Primers used in the vector construction of pLP3804

| Primer | Sequence(5’-3’) |
| --- | --- |
| P_tuf_-F | AAACTAGAAAAGATGCCCAATTCAGCAATTTAGCCT |
| P_tuf_-R | TCCTCTAGAGTCGACCTGCAGATATCTCATATGTGAAACCTCCTGTGATTT |
| Ter -F | GACTCTAGAGGATCCATGCATACCATCGATCTCGAGTGCATATTTTCGGCAATC |
| Ter-R | AAAATATAATGTCGAGGTATTCCCTCAAACTCCCTT |
| Cm-F | ACCTCGACATTATATTTT |
| Cm-R | AGCTGCCGTTATCAATGG |
| Rep-F | CCATTGATAACGGCAGCTTGTCGATCGTTGACGAAAG |
| Rep-R | GACCGGAAAACGAGTGTATTGCCCTCGCTCCTATTC |
| pUCori-F | TACACTCGTTTTCCGGTC |
| pUCori-R | GGGCATCTTTTCTAGTTT |
